# Motivation and Justice at Work: The Role of Emotion and Cognition Components of Personal and Collective Work Identity

**DOI:** 10.3389/fpsyg.2017.02307

**Published:** 2018-01-15

**Authors:** Ola Nordhall, Igor Knez

**Affiliations:** ^1^Department of Social Work and Psychology, Gävle University College, Gävle, Sweden; ^2^Department of Occupational and Public Health Sciences, Gävle University College, Gävle, Sweden

**Keywords:** work identity, personal identity, collective identity, work motivation, organizational justice

## Abstract

The aim of this study was to investigate the role of personal and collective work identity (including emotion and cognition components), in predicting work motivation (operationalized as work self-determined motivation) and organizational justice (operationalized as organizational pay justice). Digitized questionnaires were distributed by e-mail to 2905 members, teachers, of a Swedish trade union. A total of 768 individuals answered the questionnaire and by that participated in this study. Personal- compared to collective work identity was shown to positively associate with self-determined motivation accounted for by the emotion component of personal work identity. Collective compared to personal work identity was reported to positively associate with organizational pay justice accounted for by the cognition component of collective work identity. All this suggests that both work-related motivation and organizational justice might be, to some extent, accounted for by the psychological mechanisms of work identity and that, as predicted, different types of work identity, play different significant roles in predicting motivation and justice at work. More precisely, the emotion component of work identity was more pronounced in personal work-bonding relationships, and the cognitive component, of work identity in contrast, was more pronounced in collective work-bonding relationships.

## Introduction

We identify us with our work. A phenomenon labeled work identity, involving emotion and cognition processes, accounts for this type of bonding (Knez, 2016). Work identity can be divided into personal and collective identity, in general terms, involving two different foci of identification (van Knippenberg and van Schie, 2000; Millward and Haslam, 2013) that differently associate with a wide range of work- and organizational behaviors, norms and attitudes (Riketta, 2005; Riketta and Van Dick, 2005; Lee et al., 2015).

Another important factor that guides our work-related behavior and engagement (e.g., Björklund, 2001; Latham and Pinder, 2005) is work motivation, defined as “the energetic forces that initiate work-related behavior and determine its form, direction intensity and duration” (Pinder, 2008, p. 11). A further construct of great magnitude within the context of occupational work is organizational justice. It involves processes of perceived fairness and different types of interactions within the organization (Greenberg and Colquitt, 2005). Like motivation, organizational justice has been shown to associate with a wide range of crucial employee and organizational outcomes (Colquitt, 2001; Nowakowski and Conlon, 2005; Colquitt et al., 2013).

Accordingly, this paper is about work-related identity, motivation, and justice; more precisely, about the relationships between these three constructs central to different employee and organizational outcomes. We first review the research on work identity. We then review research on work motivation and organizational justice. Finally, we formulate the objectives and the associated hypotheses of the present study.

### Work identity

According to, for example, McConnell (2011) the self is a collection of multiple, context-dependent selves resulting in multiple identities. We furthermore categorize and define ourselves in terms of individual attributes, involving a personal self/identity (Hogg and Terry, 2000; Klein, 2014; Knez, 2016), and social attributes, involving a collective self/identity (Jackson et al., 2006; Knez, 2016). Given this, we can say that work identity involves two levels of abstraction as two different knowledge structures (Kihlstrom et al., 2003), namely, personal and collective work identity (Riketta, 2005; Pate et al., 2009; Miscenko and Day, 2016). In line with the self-categorization theory (e.g., Turner et al., 1987) personal work identity has the personal career or occupation as its focus of identification while collective work identity has the organization as a whole as its focus of identification (van Dick and Wagner, 2002; Riketta and Van Dick, 2005).

Thus, the foci of identification refer to variations of the identity concept “that are defined at different levels of abstraction in applied organizational context” (Millward and Haslam, 2013, p. 51). Personal and collective work identities are relatively independent of each other, meaning that having a strong identity at one level does not rule out a similarly strong identity on another level (Brewer and Gardner, 1996; Pate et al., 2009). It is furthermore suggested that these constructs might differ along the dimension of inclusiveness (Sluss and Ashforth, 2007; Miscenko and Day, 2016), resulting in different self-related definitions and interpretations (Sedikides et al., 2011; Knez, 2016). In particular, personal work identity involves classifications of “*My* profession/work” and, for example, “*I/Me* a teacher of psychology.” It articulates the psychological need to *distinguish* oneself from others (Brewer and Gardner, 1996) “in order to preserve the personal self, the personal story and its memories” (Knez, 2016, p. 3).Therefore, it is associated with individual self-related cognitions, interests, values, attitudes, motivations and behaviors (Ybarra and Trafimow, 1998; Johnson et al., 2006). By consequence, the stronger the personal work identity the stronger personal goals, preferences and needs will be (Brewer and Gardner, 1996; Ybarra and Trafimow, 1998; Ellemers et al., 2004).

In line with our psychological prerequisite to *belong* to a social group (Brewer and Gardner, 1996), collective work identity involves the *We*-descriptions “in order to be part of the collective self, the collective story and its memories” (Knez, 2016, p. 3). Accordingly, this phenomenon involves cognitions, interests, values, norms, motivations and behaviors related to the identity of a group or an organization (Ybarra and Trafimow, 1998; Johnson et al., 2006). According to social identity theory (Tajfel and Turner, 1979, 1986; see Hogg, 2012 for a review), the knowledge of belonging to a certain group includes furthermore a depersonalization of the individual self and an emotional attachment to the group/organization. Hence, the more “collectivized” an individual is the more s/he will demonstrate loyalty, acceptance, adherence to norms, values, beliefs and decisions communicated by the group/organization (Hogg and Terry, 2000; Ellemers et al., 2004; Johnson and Jackson, 2009). This suggests that normative and motivational mechanisms of the *We* will be generated by the depersonalized individual (Brewer and Gardner, 1996; Ybarra and Trafimow, 1998; Ellemers et al., 2004).

Along the lines of Knez (2014) and Knez (2016) proposed a conceptual model of the work-related self, a personal work identity including *emotion* and *cognition* categorizations of the work, based on the autobiographical memory research (e.g., Kihlstrom and Klein, 1994; Wilson and Ross, 2003; Conway and Holmes, 2004; Conway et al., 2004; Klein et al., 2004; Knez, 2017; Knez and Nordhall, 2017; Knez et al., 2017). The *emotion* component encompasses the process of work-related attachment/belongingness/closeness, and the *cognition* component involves the processes of work-related coherence, correspondence, mental time, reflection and agency. This theoretical frame of reference furthermore implies that that the work identity (encompassing above mentioned cognitive and emotional processes) is a higher order cognitive construct (Law et al., 1998; Stajkovic, 2006), a knowledge structure resulting in a personal, autobiographical work-related experience.

Knez (2016) predicted that the emotional component will precede the cognitive one in establishing personal work identity (see Knez and Eliasson, 2017 for similar argument). Finally, Knez (2016) model is general in its formulations, meaning that the psychological mechanisms accounting for the person-work bonding can be applied in both personal and collective accounts. However, in contrast to personal identity, organizational identity is more of a cognitive structure (Harquail and King, 2003) conceptualized as “a product of the dialectic relationship between collective, shared cognition on one hand and socially structured individual cognitions on the other” (Corley et al., 2006, p. 88). This suggests that the cognitive component in organizational identity, in terms of incorporation, identification and assimilation, might precede the emotional component, in terms of affective commitment, esteem and pride (Mael and Ashforth, 1992; van Knippenberg and Sleebos, 2006).

In sum, the concept of work identity used in this study involves two theoretical perspectives, levels of abstraction (in general terms foci of identification, e.g., Reichers, 1985; van Knippenberg and van Schie, 2000; Millward and Haslam, 2013): (1) personal work identity originated from a cognitive, autobiographical memory perspective (e.g., Knez, 2016); and (2) collective work identity emanated from a social identity perspective (e.g., Ashforth and Mael, 1989). As a result, we broaden the multiple focus of identification concept as a part of everyday organizational life to include personal, autobiographical work-related experiences (personal work identity). By that we suggest that personal career/occupation can be treated at both the autobiographical memory level (*individual*) and the organizational/workgroup level (*social*). This means that career/occupation at the former level “can be conceptualized as one of the life goals that we strive for and find meaning in Gini (1998), analogous with Sisyphus rolling the boulder up the hill (Camus, 1942)” (Knez, 2016, p. 2).

### Work motivation

Research on work motivation has previously shown associations between work motivation and well-being (Baard et al., 2004; Steers et al., 2004; Gagné and Deci, 2005), turnover intentions (Houkes et al., 2003), organizational commitment (Tremblay et al., 2009), and work performance and productivity (Steers et al., 2004; Rich et al., 2010), as well as to organizational and interpersonal work environment (Latham and Pinder, 2005) such as leadership (Castanheira et al., 2016), social support (Castanheira, 2016), and satisfaction (Gillet et al., 2016). Also, individual differences in perceived control, self-esteem, ego-development, defensive functioning and basic need satisfaction, have been shown to associate with work motivation (Gagné and Deci, 2005; Latham and Pinder, 2005).

One of the major theories in motivation, Self-Determination Theory (Deci and Ryan, 2000, 2002; Ryan and Deci, 2000a, 2002), has been broadly applied in the occupational work-context. According to this account (Gagné and Deci, 2005) occupational work per se has the potential to promote different psychological needs and thereby influence personal self-determined, i.e., intrinsic, motivation (Ryan and Deci, 2000b; Deci and Ryan, 2010; van den Broeck et al., 2016). Furthermore, Self-Determination Theory highlights individual rather than group-based needs and motivation and by that has the individual as its focus (Gagné and Deci, 2005; Riketta and Van Dick, 2005; Ryan and Deci, 2012; Bjerregaard et al., 2015).

Three basic psychological needs are assumed to form the foundation on which self-determined work motivation relies (Deci and Ryan, 2000; van den Broeck et al., 2016): (1) *Competence*, involving effectiveness, confidence and capacities in one's daily work and in the interplay with colleagues, managers, customers and clients (Deci and Ryan, 2000, 2008; Elliot et al., 2002; Gagné and Deci, 2005); (2) *Relatedness*, refers to feelings connected to others (colleagues, managers, customers, clients etc.) and being cared for by, and caring for, others (Deci and Ryan, 2000, 2008; Gagné and Deci, 2005); and (3) *Autonomy*, involves experiences of own behaviors and actions as expressions of the self (Deci and Ryan, 2000, 2008; Gagné and Deci, 2005).

Finally, previous findings indicate that the more one identifies with occupational work the stronger self-determined work motivation will be, and the stronger one perceives the views of one's organization to be the less one's self-determined motivation is presumed to be (Deci and Ryan, 2000; van den Broeck et al., 2016). Given all this, in this study the concept of work motivation was operationalized as the work self-determined motivation.

### Organizational justice

Organizational justice has been shown to associate with both individual and organizational outcomes (Greenberg, 2011; Colquitt et al., 2013), such as work performance (Wang et al., 2015), job satisfaction (Greenberg, 2011), organizational commitment (López-Cabarcos et al., 2015), counterproductive behavior Cremer (2006), turnover intention (Aryee et al., 2002), organizational citizenship behavior (Blader and Tyler, 2009), health related factors like sick leave, stress related problems, cardiovascular problems, burnout and emotional exhaustion (Greenberg, 2010; Ndjaboué et al., 2012; Robbins et al., 2012; Piccoli and De Witte, 2015), and anxiety and depression (Spell and Arnold, 2007).

Several predictors of organizational justice have also been indicated, such as trust in the supervisor or organization (Aryee et al., 2002), perceived equity and equality within the organization (Colquitt et al., 2005), needs (see Greenberg and Cohen, 1982), job security, complexity and status (Ambrose and Arnaud, 2005) moral and ethical standards (Folger et al., 2005), perceived organizational support (Bies, 2005), and expectations (Colquitt et al., 2005).

This construct has furthermore been shown to comprise four independent dimensions (Colquitt, 2001; Ambrose and Arnaud, 2005; Colquitt and Shaw, 2005; Greenberg and Colquitt, 2005; Ambrose and Schminke, 2009; Nicklin et al., 2014):

***Procedural*** justice involves perceptions of fairness related to organizational procedures (Colquitt et al., 2005; Greenberg, 2011). One central issue is the opportunity to “voice,” i.e. to express one's opinions and concerns (Thibaut and Walker, 1975). Also, consistency, correctness, lack of bias, and accuracy foster this dimension (Leventhal, 1980).

***Distributive*** justice involves perceptions of fairness related to the distribution of outcomes such as money, rewards, and time (Colquitt et al., 2005; Greenberg, 2011). It is fostered when outcomes are consistent with respect to equity and equality (Adams, 1965; Leventhal, 1980) and when personal effort-outcome ratios match effort-outcome ratios of significant others (Adams, 1965).

***Interpersonal*** justice involves perceptions of the supervisor's conduct, in terms of being courteous. Even though decisions might entail negative consequences for the recipient, they are still perceived as fair if the individual recognizes him/herself to be respectfully treated by the supervisor (Greenberg, 1990, 1993).

***Informative*** justice involves the amount, quality and timing of the information received by the employee (Greenberg, 1993). In addition, there should be regular opportunities to receive relevant and adequate explanations of, and arguments for (Bobocel and Zdaniuk, 2005), for example, payment decisions (Andersson-Stråberg et al., 2007).

Previous research strongly suggests including all four justice dimensions in studies related to organizational context issues (Bies, 2005). However, since this phenomenon emanates from the organizational work-environment and thus has the organization, i.e., the collective as its focus (Riketta and Van Dick, 2005), it is reasonable to assume that organizational related issues like organization-related identity, loyalty, shared values and preferences, may be more critical for the organizational justice (Cremer, 2006; Blader and Tyler, 2009). Finally, in this study the concept of organizational justice was operationalized as organizational pay justice, because it originates from an organizational framework (Greenberg and Colquitt, 2005; Andersson-Stråberg et al., 2007) and by that might link with collective work identity (see Present Study below for this line of reasoning).

### Present study

In line with self-categorization theory (e.g., Turner et al., 1987) and the general notion of foci of identification and its associated effects, the relationship between work identity and other work-related constructs might be stronger when foci of categorization between the constructs match; thus, when both the type of identity (predictor) and the outcome (criterion variable) are conceptually related (van Dick and Wagner, 2002; Riketta and Van Dick, 2005; Olkkonen and Lipponen, 2006).

Within the frameworks of social identity theory (Tajfel and Turner, 1979, 1986; see Hogg, 2012 for a review), the two predominating foci of identification (and outcomes) have been work-group (relational level) and organization (collective level), both involving a social focus of identification. This means that the personal focus of identification has to a large extent been neglected in previous research (van Dick and Wagner, 2002; Riketta, 2005; Riketta and Van Dick, 2005). Given this, the aim of the present study was to explicitly test the general notion of foci of identification and its associated effects within and beyond the social focus of identification. This was done by investigating *both* the *personal* and the *collective* focus of identification in relation to work-related motivation and justice.

By consequence and given that personal and collective work identity might tap psychological processes at different levels of abstraction (Haslam et al., 2000; Sluss and Ashforth, 2007), we hypothesized that these constructs will associate differently with work-related motivation and justice.

It has, for example, been reported that collective work identity is not associated with individual internal motivation (van Knippenberg and van Schie, 2000), and that a positive association between collective work identity and organizational-related motivation and behaviors is stronger in a collectivistic vs. an individualistic organizational culture (Lee et al., 2015). Also, the more one identifies with one's occupational work tasks (personal/individual identification) the stronger one's basic need satisfaction is, and the stronger one identifies with one's organization (collective identification), the weaker one's self-determined motivation is (van den Broeck et al., 2016).

The two different structures of work identity have been, furthermore, related to different motivational (van Knippenberg and van Schie, 2000; Ellemers et al., 2004; Johnson et al., 2010; He et al., 2014) and attitudinal (Ybarra and Trafimow, 1998) outcomes. Previous studies have, however, largely addressed the motivational issues on behalf of the organizational collective as opposed to the personal motivation to perform well. Also, the focus of identification has to a large extent been framed in terms of social identity; thus, relational and/or collective work identity (van Knippenberg, 2000; van Dick and Wagner, 2002; Bjerregaard et al., 2015).

Most previous findings on the relationships between work identity and organizational justice have, in line with the group-engagement model (Tyler and Blader, 2003; Blader and Tyler, 2009), focused on justice as a decisive predictor of different levels of work identity. This was done to clarify the role of identity-based information incorporated in organizational justice (Lipponen et al., 2011), as well as the mediating role of work identity in relation to the effects of organizational justice on different outcomes (Olkkonen and Lipponen, 2006; Cho and Treadway, 2011; He et al., 2014). Finally, interaction effects of social identity and organizational justice dimensions on different work-related outcomes have additionally been investigated (Cremer, 2006; Lipponen et al., 2011; Smith, 2012).

Regarding the relationship between work identity and organizational justice, it is suggested that the self-constitutes one of the predicting foundations for self-reported justice (Skitka, 2003). Several findings have indicated that this association primarily concerns collective work identity (Olkkonen and Lipponen, 2006; Blader and Tyler, 2009; Cho and Treadway, 2011; Ehrhardt et al., 2012; He et al., 2014).

Despite the previous research on work-related motivation and justice no previous research has, as far as we know, investigated the role of personal and collective work identity in predicting these constructs. Accordingly, and in line with the general notion of foci of identification and its associated effects (van Dick and Wagner, 2002; Riketta and Van Dick, 2005; Olkkonen and Lipponen, 2006) the aim of the present study was to investigate the relationships between personal and collective work identity, as predictors, and work-related motivation and justice as criterion variables. This involves both matching variables and two levels of explanation; that is, a link between: (1) *Personal* work identity and work-related *Self*-determined motivation (personal level of explanation); and (2) *Collective* work identity and *Organizational* pay justice (collective level of explanation).

### Hypotheses

Given the above, we predicted:

***Hypothesis 1***. In accordance with Deci and Ryan (2000) and van den Broeck et al. (2016), the more one identifies with occupational work the stronger self-determined motivation one will be subjected to. Accordingly, the association might be stronger when identity and motivation conceptually match (Riketta and Van Dick, 2005; Olkkonen and Lipponen, 2006). Hence, we predicted a stronger positive association of personal work identity 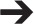 work self-determined motivation than collective work identity 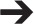 work self-determined motivation.

***Hypothesis 2***. In line with Knez (2016) and Knez and Eliasson (2017), we predicted that emotion compared to cognition component of personal work identity would show a stronger positive association with work self-determined motivation.

***Hypothesis 3***. Given that organizational pay justice (Greenberg and Colquitt, 2005; Andersson-Stråberg et al., 2007) emanates from an organizational framework, and thus has the same level of explanation as collective work identity, we hypothesized a stronger positive association of collective work identity 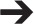 organizational pay justice than personal work identity 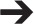 organizational pay justice. This suggests that organizational compared to individual work-related issues might be more central to organizational justice (Tajfel and Turner, 1979; Cremer, 2006; Blader and Tyler, 2009).

***Hypothesis 4***. In line with some previous research suggesting organizational identity to be more of a cognitive structure of shared collective and individual cognitions (Harquail and King, 2003; Haslam and Ellemers, 2005; Corley et al., 2006), we predicted that cognition compared to emotion component of collective work identity would show a stronger positive association with organizational pay justice.

## Methods

### Participants

Digitized questionnaires were distributed by e-mail to 2905 members of the Swedish trade union “The National Union of Teachers” (in Swedish “Lärarnas Riksförbund”) working in the south and middle part of Sweden, during April and May 2016. A total of 768 questionnaires were returned, with a response rate of 26%. Ninety-nine percent of the participants had an educational function, that is, they worked as teachers or similar. Also, the participants worked within the municipal sector (92.1%), had university studies as their highest level of education (68%), were in permanent employment (95.2%), had full-time jobs (80.5%) and were female (75.5%). Mean age was 46.3 (*SD* = 10.07, range 24–67) and mean employment time within the organization was 14 years (*SD* = 10.2).

### Measures

#### Work motivation

Work motivation was measured by “Work Extrinsic and Intrinsic Motivation Scale,” WEIMS (Tremblay et al., 2009), applied in several previous studies (Peklar and Boštjančič, 2012; Stoeber et al., 2013; Jayaweera, 2015; Saltson and Nsiah, 2015). WEIMS is an 18-item measure theoretical grounded in self-determination theory (Deci and Ryan, 2000; Ryan and Deci, 2000a; Gagné and Deci, 2005). Participants responded to the WEIMS-items regarding the question “Why do you do your work?” on a 5-point Likert scale, ranging from 1 (“Does not correspond at all”) to 5 (“Corresponds exactly”). WEIMS contains six subscales measuring: (a) Intrinsic motivation (e.g., “Because I derive much pleasure from learning new things”); (b) Integrated regulation (e.g. “Because it is part of the way in which I have chosen to live my life”); (c) Identified regulation (e.g., “Because I chose this type of work to attain my career goals”); (d) Introjected regulation (e.g., “Because I want to be a “winner” in life”); (e) External regulation (e.g., “For the income it provides me”); (f) Amotivation (e.g., “I don't know, too much is expected of us”). In order to obtain a measure of the participants' overall work motivation, the subscales a-f were aggregated into an overall “Work self-determined index,” W-SDI (Tremblay et al., 2009). The response scale has a range of ±24. The positive part of the scale indicates self-determined work motivation, while the negative one indicates non-self-determined work motivation (Vallerand and Ratelle, 2002; Tremblay et al., 2009). Good psychometric properties have been reported for this measure, with a Cronbach alpha (α) of 0.84 (Tremblay et al., 2009). In the current study the Cronbach alpha (α) of work self-determined motivation was 0.74, indicating acceptable internal consistency (DeVellis, 2003).

#### Organizational pay justice

In order to measure organizational pay justice as a construct derived from the more general concept of organizational justice, we used Colquitt's (2001) 20-item measure (Colquitt and Shaw, 2005; see also Greenberg, 2011), measuring Procedural pay justice (e.g., “You have been able to express your views and feelings during the pay procedures”); Distributive pay justice (e.g., “Your salary reflects the effort you have put into your work”); Interpersonal pay justice (e.g., “The pay setting manager has treated you in a polite manner”); Informative pay justice (e.g., “The pay setting manager has explained the procedures thoroughly”). Participants responded to the statements on a 5-point Likert scale, ranging from 1 (“To a very small extent”) to 5 (“To a very large extent”). Previous studies have shown good fit for the four-factor model of this measure with good psychometric properties (Colquitt, 2001; Andersson-Stråberg et al., 2007). In the present study, the four dimensions of organizational pay justice showed the following Cronbach alpha values (α): procedural pay justice 0.91; distributive pay justice 0.95; interpersonal pay justice 0.93; and informative pay justice 0.94. This indicates very good internal consistency for organizational pay justice dimensions (DeVellis, 2003).

#### Work identity

In order to measure personal work identity we used a measure suggested by Knez (2014, 2016) and Knez and Eliasson (2017). It includes 10 statements measuring emotion and cognition components of personal work identity: Emotion (“I am keenly familiar with my work”; “I miss it when I'm not there.”; “I have strong ties to my work.”; “I am proud of my work.”; “It is a part of me.”); Cognition (“I have had a personal contact with my work over a long period.”; “There is a link between my work and my current life.”; “I can travel back and forth in time mentally to my work when I think about it.”; “I can reflect on the memories of my work”; “Thoughts about my work are part of me.”). Participants were asked to respond to the statements on a 5-point Likert-scale ranging from 1 (completely disagree) to 5 (completely agree). In the present study, the Cronbach alpha (α) values were 0.86 for personal work identity and 0.75 for emotion and 0.84 for cognition component respectively, indicating acceptable-good internal consistency (DeVellis, 2003).

In addition, we examined the construct validity of the personal work identity construct with a confirmatory factor analysis, including a second-order factor representing a general personal work identity construct, with cognitive and emotional components as first-order factors. According to Byrne (2016) the model showed acceptable data fit of Chi2 = 188.57, *df* = 28 (*p* = 0.000), CFI = 0.95 and RMSEA = 0.08.

Collective work identity was measured by the “Identification with a Psychological Group Scale” (Mael and Ashforth, 1992; Mael and Tetrick, 1992; Riketta, 2005), theoretically grounded in Social Identity Theory (Tajfel and Turner, 1979, 1986) and the Self-Categorization Theory (Hogg and Terry, 2000). This measure includes six statements with a 5-point Likert scale, ranging from 1 (completely disagree) to 5 (completely agree). Based on the conceptual model of Knez (2014, 2016) that distinguishes between an emotion and a cognition component of the work-related personal and collective self (Jackson et al., 2006; Knez, 2016), the items of the “Identification with a Psychological Group Scale” (Mael and Ashforth, 1992), was, besides being indexed, categorized into an Emotion component (item 1 = “When someone criticizes my organization, it feels like a personal insult”; 5 = “When someone praises the organization, it feels like a personal compliment”; 6 = “If a story in the media criticized the organization, I would feel embarrassed”), and a Cognition component (item 2 = “I am very interested in what others think about the organization”; 3 = “When I talk about the organization, I usually say ‘we’ rather than ‘they”’; 4 = “This organizations' successes are my successes”). This was done in line with Mael and Ashforth's (1992) suggestions.

The following Cronbach alphas (α) were reported in the present study: 0.87 for collective work identity, and 0.78 for emotional and 0.77 for cognitive component, indicating good internal consistency (DeVellis, 2003). We also, as above, examined the construct validity of the collective work identity construct, including a second-order factor representing a general collective work identity, with cognitive and emotional components as first-order factors. This model showed acceptable data fit of Chi2 = 64.09, *df* = 7 (*p* = 0.000), CFI = 0.97 and RMSEA = 0.10 (Byrne, 2016).

### Procedure

Chairpersons of 11 municipal associations of the Swedish trade union The National Union of Teachers were contacted and informed of the purpose of the present study. They were asked to invite their union members to participate in the present study, of which they approved. Due to Swedish juridical restrictions that do not permit a chairperson of a union to distribute individual e-mail addresses of the members, a web-link to the questionnaire was distributed to the members by the chairpersons. The questionnaires were accompanied by a covering letter that described the purpose of the study and informed the participants that completion of the questionnaire was taken as an indication of their consent to participate in the present study, and that this was voluntary and that confidentiality and anonymity was assured. After completion of the questionnaire the participants were asked to fill in their name and address if they wanted to receive a cinema ticket as compensation for their participation. They were informed that nobody but the researchers of the present study would have access to their names and addresses.

Finally, an ethical application was reviewed and approved by the Swedish regional ethical committee of Uppsala University (Dnr 2015/423).

### Design and analyses

In line with the four hypotheses, four types of multiple hierarchical regression analyses were performed in order to investigate the role of personal and collective work identity in predicting motivation and justice at work:

1. Personal- and collective work identity (predictors) and work self-determined motivation (criterion variable)
2. Emotion- and cognition component of personal work identity (predictors) and work self-determined motivation (criterion variable)
3. Personal- and collective work identity (predictors) and organizational pay justice (including procedural, distributive, interpersonal, informative dimensions as criterion variables)
4. Emotion- and cognition component of collective work identity (predictors) and organizational pay justice (including procedural, distributive, interpersonal, informative dimensions as criterion variables)

In all four analyses, we controlled additionally for the effects of: *monthly income*; *school sector* (public vs. private); *years of employment*; and *permanent employment* (no vs. yes). These variables have previously been addressed as potential confounders, related to work self-determination motivation (Amabile et al., 1994; Vansteenkiste et al., 2007; Howard et al., 2016; Ryan and Deci, 2017) and organizational pay justice (Ambrose and Schminke, 2003; Andersson-Stråberg et al., 2007; Fortin, 2008). In order to specify a fixed order of entry to control for the effects of potential confounders, in the four multiple hierarchical regression analyses, the covariates were entered in step one and predictors in step two.

## Results

First, we report the bivariate correlations, *N*, mean and standard deviation statistics for all variables included in the regression analyses (see Table 1). Second, we report the results obtained in accordance with our hypotheses and the types of regression analyses associated with each one of the four hypotheses respectively (see section Design and Analyses). None of the statistical analyses below were subjected to multicollinearity effects, showing Tolerance values of >0.10, range 0.446–0.934 and all VIF (variance inflation factor) <10, range 1.070–2.242 (see Myers, 1990; Menard, 1995; Tabachnick and Fidell, 2012).

**Table 1:** Bivariate correlations (r), *N*, mean (*M*), and standard deviation (*SD*) statistics for all variables included in regression analyses.

**Variables**	***N***	***M***	***SD***	**1**	**2**	**3**	**4**	**5**	**6**	**7**	**8**	**9**	**10**	**11**	**12**	**13**	**14**
1 PWI	767	3.62	0.70														
2 E-PWI	767	3.62	0.71	0.874^**^													
3 C-PWI	767	3.61	0.86	0.918^**^	0.610^**^												
4 CWI	767	2.78	0.91	0.294^**^	0.291^**^	0.243^**^											
5 E-CWI	767	2.59	0.99	0.267^**^	0.248^**^	0.232^**^	0.934^**^										
6 C-CWI	767	2.97	0.97	0.282^**^	0.294^**^	0.220^**^	0.931^**^	0.739^**^									
7 WSDM	766	4.97	6.05	0.492^**^	0.588^**^	0.322^**^	0.180^**^	0.121^**^	0.216^**^								
8 P-OPJ	767	2.50	0.94	0.161^**^	0.185^**^	0.112^**^	0.345^**^	0.310^**^	0.333^**^	0.265^**^							
9 D-OPJ	767	2.18	1.13	0.076^*^	0.116^**^	0.029	0.142^**^	0.121^**^	0.144^**^	0.197^**^	0.523^**^						
10 Int.-OPJ	767	3.96	1.07	0.059	0.114^**^	0.003	0.225^**^	0.191^**^	0.229^**^	0.241^**^	0.588^**^	0.383^**^					
11 Inf.-OPJ	767	3.08	1.18	0.089^*^	0.128^**^	0.041	0.225^**^	0.195^**^	0.225^**^	0.226^**^	0.701^**^	0.457^**^	0.667^**^				
12 Monthly income	768	31909.59	4975.10	0.159^**^	0.176^**^	0.116^**^	0.074^*^	0.047	0.091^*^	0.069	0.141^**^	0.305^**^	0.158^**^	0.150^**^			
13 School sector	768	na.^***^	na.^***^	−0.032	−0.042	−0.018	0.009	0.016	0.000	0.009	0.005	−0.028	−0.092^*^	0.083^*^	−0.149^**^		
14 Years of employment	768	13.97	10.22	0.063	0.063	0.052	−0.048	−0.036	−0.055	−0.038	−0.078^*^	−0.002	−0.049	−0.001	0.346^**^	−0.239^**^	
15 Permanent employment	768	na.^***^	na.^***^	0.118^**^	0.160^**^	0.062	0.071	0.048	0.084^*^	0.038	0.028	0.003	0.088^*^	0.070	0.275^**^	−120^**^	0.206^**^

*Personal work identity (PWI); emotion (E-) and cognition (C-) component of PWI; collective work identity (CWI); emotion (E-) and cognition (C-) component of CWI; work self-determined motivation (WSDM); procedural- (P-); distributive (D-); interpersonal (Int.-); informative- (Inf.-) organizational pay justice (-OPJ); monthly income; school sector; years of employment; permanent employment*.

*** *na., not applicable due to categorical data*.

** *Correlation significant at the 0.01 level (2-tailed)*.

* *Correlation significant at the 0.05 level (2-tailed)*.

### Personal- and collective work identity and work self-determined motivation

Together, personal- and collective work identity predicted work self-determined motivation, with an additional explained variance of 24% after controlling for the four potentially confounding variables. As predicted (*Hypothesis 1)*, personal compared to collective work-related identity was shown to positively associate with work self-determined motivation (see Table 2; see also Appendix 1 for the first step regression statistics).

**Table 2:** Relation between personal- (PWI) and collective (CWI) work identity and work self-determined motivation after controlling for the four covariates (monthly income, school sector, years of employment, permanent employment).

***R^2^ change***	**Beta (β)**	***df***	***F change***	***T***	***p***
0.24		2,758	120.33		0.000
	0.49 PWI			14.55	0.000
	0.03 CWI			1.00	0.316

### Emotion- and cognition component of personal work identity and self-determined motivation

Together, emotion- and cognition components of personal work identity predicted work self-determined motivation, with an additional explained variance of 35% after controlling for the four potentially confounding variables. In agreement with *Hypothesis 2*, emotion- compared to cognition component of personal work identity was shown to positively associate with work self-determined motivation (see Table 3; see also Appendix 1 for the first step regression statistics).

**Table 3:** Relation between emotion- (E-) and cognition (C-) component of personal work identity (PWI) and work self-determined motivation after controlling for the four covariates (monthly income, school sector, years of employment, permanent employment).

***R^2^ change***	**Beta (β)**	***df***	***F change***	***t***	***p***
0.35		2,758	203.29		0.000
	0.64 E-PWI			16.99	0.000
	−0.06 C-PWI			−1.60	0.109

### Personal- and collective work identity and organizational pay justice

Together, personal- and collective work identity predicted all organizational pay justice dimensions, with an additional explained variance of 11.1% in procedural pay justice, 1.3% in distributive pay justice, 4.2% in interpersonal pay justice and 4.4% in informative pay justice, respectively, after controlling for the four potentially confounding variables. Consistent with *Hypothesis 3* collective- compared to personal work identity was shown to positively associate with all organizational pay justice dimensions (see Table 4; see also Appendix 2 for the first step regression statistics).

**Table 4:** Relation between personal- (PWI) and collective (CWI) work identity and organizational pay justice dimensions after controlling for the four covariates (monthly income, school sector, years of employment, permanent employment).

**Pay justice dimension**	***R^2^ change***	**Beta (β)**	***df***	***F change***	***t***	***p***
Procedural	0.11		2.760	48.59		0.000
		0.05PWI			1.52	0.128
		0.31CWI			8.90	0.000
Distributive	0.01		2.760	5.80		0.003
		0.00PWI			0.048	0.962
		0.12CWI			3.25	0.001
Interpersonal	0.04		2.760	17.60		0.000
		−0.03PWI			−0.83	0.409
		0.21CWI			5.87	0.000
Informative	0.04		2.760	18.01		
		0.01PWI			0.13	0.895
		0.21CWI			5.71	0.000

### Emotion and cognition component of collective work identity and organizational pay justice

Together, emotion and cognition components of collective work identity predicted the organizational pay justice dimensions with an additional explained variance of 11% in procedural pay justice, 1.4% in distributive pay justice, 4.3% in interpersonal and 4.5% in informative pay justice, respectively, after controlling for the four potentially confounding variables. In line with *Hypothesis 4*, cognition vs. emotion component of collective work identity showed a stronger positive relationship with organizational pay justice, with exception for the distributive justice dimension. In other words, the cognition component of collective work identity accounted for a stronger association with three dimensions of organizational pay justice than did the emotion component of collective work identity (see Table 5; see also Appendix 2 for the first step regression statistics).

**Table 5:** Relation between emotion- (E-) and cognition (C-) component of collective work identity (CWI) and organizational pay justice dimensions after controlling for the four covariates (monthly income, school sector, years of employment, permanent employment).

**Pay justice dimension**	***R^2^ change***	**Beta (β)**	***df***	***F change***	***T***	***P***
Procedural	0.11		2.760	47.51		0.000
		0.15 E-CWI			2.99	0.003
		0.20 C-CWI			4.10	0.000
Distributive	0.01		2.760	5.86		0.003
		0.05 E-CWI			0.93	0.352
		0.08 C-CWI			1.54	0.124
Interpersonal	0.04		2.760	17.88		0.000
		0.06 E-CWI			1.18	0.240
		0.16 C-CWI			3.09	0.002
Informative	0.05		2.760	18.36		0.000
		0.07 E-CWI			1.44	0.151
		0.15 C-CWI			2.92	0.004

## Discussion

The aim of this study was to investigate the role of personal and collective work identity (including emotion and cognition components) in predicting motivation and justice at work, in a Swedish teacher sample (*N* = 768). In line with a general notion of focus of identification and its associated effects (van Dick and Wagner, 2002; Riketta and Van Dick, 2005; Olkkonen and Lipponen, 2006) and our predictions, we showed that both personal and collective work identity played significant, but different roles in predicting work-related motivation and organizational pay justice respectively. These relationships were, furthermore, not influenced by any of the previously suggested confounders of monthly income, school sector, years of employment, and permanent employment (Amabile et al., 1994; Ambrose and Schminke, 2003; Andersson-Stråberg et al., 2007; Vansteenkiste et al., 2007; Fortin, 2008; Howard et al., 2016; Ryan and Deci, 2017).

More precisely and concerning *Hypotheses 1* and *2*, it was shown that personal compared to collective work identity positively associated with work self-determined motivation, accounted for by the emotion component of personal work identity. Also, this is in accordance with previous research indicating that occupational task identity (i.e., personal identification) positively predicts all of the three psychological needs; competence, relatedness and autonomy, which may function as the basis of self-determined work motivation (Deci and Ryan, 2000; van den Broeck et al., 2016). Furthermore, the present results are in accordance with findings that collective (organizational) identity is neither positively nor negatively associated with satisfaction of personal motivators and needs, i.e., factors specific to the individual employee (Haslam et al., 2000), for example that collective identity is not associated with individual internal motivation (van Knippenberg and van Schie, 2000). By this, one might suppose that the personal- compared to collective work identity constitutes a crucial foundation on which satisfaction of the psychological needs (Deci and Ryan, 2000) is based.

The results related to Hypothesis 2 are in line with Knez (2014) and Knez and Eliasson (2017), showing that emotion component precedes the cognitive one in personal identity and by that accounts for its effects. This implies that the extent to which work motivation is self-determined, i.e. that employees are autonomously motivated in terms of pure satisfaction and interest, is stronger related to processes of personal work-related attachment/belongingness/closeness (emotion component), compared to cognitive processes of coherence, correspondence, mental time, reflection and agency (Knez, 2014, 2016). In general, this is also in line with findings showing that emotion may regulate intrinsic psychological processes (Gross, 2010), and enhance retention and recall in autobiographical memory (Canli et al., 2000; Phelps, 2006; Knez et al., 2017); a type of memory that grounds identity construction (Conway, 2005; Knez, 2017; Knez and Nordhall, 2017).

Regarding *Hypotheses 3* and *4*, collective compared to personal work identity was reported to positively associate with organizational pay justice accounted for by the cognition component of collective work identity. Firstly, this is in line with previous findings showing that the individual self-concept is not associated with organizational pay justice, while the collective self-concept is positively associated with different organizational pay justice dimensions (Johnson et al., 2006; Ehrhardt et al., 2012). This indicates that collective contrary to personal work identity has stronger associations with whether one judges the outcomes, procedures, interpersonal treatment and communication in an organizational context to be more or less fair. Furthermore, the results related to Hypothesis 3 are in line with the Accessible Identity Model which posits that how people define and perceive justice depends on which aspect of the self (social, personal etc.) dominates the working self-concept. Thus, in order to understand perceptions and reasons about justice one has to know the accessibility of different self-aspects, indicating the self as one of the predicting foundations of justice perceptions (Skitka, 2003).

Secondly, the results related to Hypothesis 4 are in accordance with previous studies indicating that in contrast to personal- collective (organizational) work identity is more of a cognitive structure (Harquail and King, 2003). Thus, how fair one judges the outcomes, procedures, interpersonal treatment and communication in an organizational context to be is strongly related to processes of incorporation, identification and assimilation making up the cognitive bond to the organization, compared to processes of affective commitment, esteem and pride making up the emotional bond to the organization (Mael and Ashforth, 1992; van Knippenberg and Sleebos, 2006).

Finally, our results are in general consistent with the suggestion that when focus of identity match the focus of outcome, the association will be stronger compared to when the two foci do not match (van Dick and Wagner, 2002; Riketta and Van Dick, 2005; Olkkonen and Lipponen, 2006). Given this, our findings extend the results of Riketta and Van Dick (2005) and Olkkonen and Lipponen (2006) by showing that the notion of foci of identification and its associated effects also yields both individual and collective work identity.

## Conclusion

Our results highlight the importance of person-work bonding by suggesting that motivation and justice at work might be, to some extent, accounted for by the psychological mechanisms of work identity. We have also reported that these relationships were not influenced by the previously indicated confounders of monthly income, school sector, years of employment, and permanent employment (Amabile et al., 1994; Ambrose and Schminke, 2003; Andersson-Stråberg et al., 2007; Vansteenkiste et al., 2007; Fortin, 2008; Howard et al., 2016; Ryan and Deci, 2017). Hence, we have shown that the links between work identity and other work-related constructs are stronger when foci of categorization between the constructs match. As a result, when both the type of identity and the outcome are conceptually related the association will be stronger; however, differing across personal and collective work identity respectively. In other words, the emotion component of work identity was more pronounced in personal work-bonding relationships, and the cognitive component of work identity, in contrast, was more pronounced in collective work-bonding relationships.

Practically this implies that emotional personal- and cognitive collective work identity constitute psychological resources for the teachers in their everyday work setting (Bakker and Demerouti, 2008; Bakker and Bal, 2010; Choochom, 2016). More precisely, when teachers *feel more* and *think less* about their *personal* work-bonding, they are more self-determined motivated and so may have stronger need satisfaction regarding competence, relatedness and autonomy (Deci and Ryan, 2000; van den Broeck et al., 2016). By contrast, when teachers *think more* and *feel less* about their *collective* work-bonding, they experience a stronger sense of justice regarding the procedural, interpersonal and informative aspects of their payment.

## Ethics statement

This study was carried out in accordance with the recommendations of APA with written informed consent from all subjects. The project has been approved by the local ethical board, at the University of Uppsala, Sweden (Dnr 2015/423).

## Author contributions

ON collected the data, did the data analyses and wrote all manuscript sections together with IK.

### Conflict of interest statement

The authors declare that the research was conducted in the absence of any commercial or financial relationships that could be construed as a potential conflict of interest.
