# Supplementary material for: Motivation and Justice at Work: The Role of Emotion and Cognition Components of Personal and Collective Work Identity
Source: Front Psychol. 2018 Jan 15;8:2307. doi: 10.3389/fpsyg.2017.02307 (PMC5775288; doi:10.3389/fpsyg.2017.02307)
Supplement: Supplementary file 1 [file DataSheet1.docx]

**Appendix 1**

**Statistics for the first step of the hierarchical regression analysis involving four covariates [monthly income (MI), school sector (SC), years of employment (YE), permanent employment (PE)] and criterion variable of work self-determined motivation. See Table 2 and 3 for the main (second step) results.**

| Step | R^2^ | Beta (β) | df | F | *t* | *p* |
| --- | --- | --- | --- | --- | --- | --- |
| 1 | .01 |  | 4, 760 | 1.89 |  | .111 |
|  |  | .09 MI |  |  | 2.18 | .030 |
|  |  | .01 SC |  |  | .21 | .833 |
|  |  | -.07 YE |  |  | -1.82 | .070 |
|  |  | .03 PE |  |  | .80 | .422 |

**Appendix 2**

**Statistics for the first step of the hierarchical regression analysis involving four covariates [monthly income (MI), school sector (SC), years of employment (YE), permanent employment (PE)], and the criterion variables of procedural, distributive, interpersonal and informative pay justice. See Table 4 and 5 for the main (second step) results.**

| Step | R^2^ | Beta (β) | df | *F* | *t* | *p* |
| --- | --- | --- | --- | --- | --- | --- |
| Step 1 Procedural | .20 |  | 4, 762 | 7.51 |  | .000 |
|  |  | .19 MI |  |  | 4.85 | .000 |
|  |  | -.00 SC |  |  | -.021 | .983 |
|  |  | -.14 YE |  |  | -3.72 | .000 |
|  |  | .01 PE |  |  | .148 | .883 |
| Step 1 Distributive | .11 |  | 4,762 | 23.82 |  | .000 |
|  |  | .36 MI |  |  | 9.72 | .000 |
|  |  | -.01 SC |  |  | -.28 | .779 |
|  |  | -.11 YE |  |  | -3.06 | .002 |
|  |  | -.07 PE |  |  | -2.07 | .038 |
| Step 1 Interpersonal | .05 |  | 4,762 | 9.84 |  | .000 |
|  |  | .18 MI |  |  | 4.63 | .000 |
|  |  | -.09 SC |  |  | -2.55 | .011 |
|  |  | -.15 YE |  |  | -3.76 | .000 |
|  |  | .06 PE |  |  | 1.54 | .124 |
| Step 1 Informative | .03 |  | 4,762 | 6.37 |  | .000 |
|  |  | .16 MI |  |  | 4.02 | .000 |
|  |  | -. 08 SC |  |  | -2.04 | .042 |
|  |  | -. 08 YE |  |  | -2.07 | .039 |
|  |  | .04 PE |  |  | .93 | .353 |
